# Comparative effectiveness of alloplastic and biologic grafts in maxillary sinus augmentation: a systematic review

**DOI:** 10.1038/s41405-026-00435-y

**Published:** 2026-05-07

**Authors:** Jonathan Varghese Thomas, Santosh Martande, Joshua Thomas Meenathathil, Bhushan Bhagat, Swathi PV, Shambhavi Thakur, Krishna Suryawanshi, Nomita Yein

**Affiliations:** 1 Department of Periodontology, Dr. D. Y. Patil Dental College and Hospital, Dr. D. Y. Patil Vidyapeeth, Pimpri, Pune, India; 2 Private Practitioner, Scientific Dental Clinic, Edappally, Kochi, Kerala India; 3 Department of Oral and Maxillofacial Surgery, Dr. D. Y. Patil Dental College and Hospital, Dr. D. Y. Patil Vidyapeeth, Pimpri, Pune, India; 4 Department of Orthodontics and Dentofacial Orthopedics, Dr. D. Y. Patil Dental College and Hospital, Dr. D. Y. Patil Vidyapeeth, Pimpri, Pune, India

**Keywords:** Dental implants, Dental implants

## Abstract

**Background:**

Maxillary sinus augmentation is a well-established procedure to increase posterior maxillary bone volume for implant placement. Various graft materials are used, including autografts, allografts, xenografts, and alloplasts, but the clinical performance of alloplasts remains debated.

**Methodology:**

A systematic review was performed following PRISMA guidelines to compare histological, histomorphometric, radiographic, and clinical outcomes of sinus lift procedures using different grafts. Electronic searches were conducted in eight databases. Eligible studies were human clinical trials (RCTs, cohorts) comparing at least two graft materials and reporting new bone formation, graft resorption, implant survival, or complications. Eighteen studies met the inclusion criteria. Risk of bias was assessed using Cochrane RoB 2.0 and ROBINS I V2 tool.

**Results:**

Alloplastic grafts demonstrated comparatively lower new bone formation than autogenous and allogeneic grafts, and were generally inferior to xenografts in terms of regenerative potential. They exhibited higher residual graft content, suggesting slower resorption when compared with biologic grafts. In contrast, xenografts and allografts showed more favorable integration. Volumetric outcomes indicated that alloplastic grafts provided better dimensional stability than autografts but were less effective than xenografts in maintaining graft volume and space. Radiographic findings further supported superior space maintenance with xenografts compared to alloplastic grafts. Implant survival rates were high across all graft types; however, biologic grafts demonstrated relatively improved success and lower marginal bone loss compared with alloplastic grafts.

**Conclusion:**

Autografts demonstrated the highest effectiveness in maxillary sinus augmentation, followed by xenografts and alloplastic grafts, indicating a comparatively lower regenerative potential of alloplastic materials. Nevertheless, alloplastic grafts may still be considered a viable alternative in selected clinical scenarios, particularly where minimizing donor-site morbidity or reducing immunological and infection-related risks is a priority. High-quality, long-term randomized trials are needed to confirm these findings and optimize graft selection.

## Introduction

Due to limited bone height and quality brought on by alveolar ridge resorption and maxillary sinus pneumatization after tooth loss, placing dental implants in the posterior maxilla frequently poses a clinical challenge [1]. To address this anatomical limitation, maxillary sinus floor elevation, commonly referred to as sinus lift surgery, has become a widely accepted technique to augment vertical bone height and enable successful implant rehabilitation [2, 3].

Numerous bone graft materials have been used in procedures involving sinus augmentation, each with distinct biological properties and clinical outcomes. Autogenous bone, traditionally considered the gold standard, possesses osteoconductive, osteoinductive, and osteogenic properties. However, its use is often limited by donor-site morbidity, limited volume availability, and rapid resorption rates [4]. Xenografts and allografts offer off-the-shelf alternatives with good volume stability, though concerns regarding immunogenicity, disease transmission, and slower bone remodeling remain [5].

Artificial bone substitutes such as hydroxyapatite (HA), β-tricalcium phosphate (β-TCP), and bioactive glass, known as alloplastic grafts, have become more popular in recent years because of their biocompatibility, lack of risk of disease transmission, and predictable resorption rates [6]. These materials are primarily osteoconductive and serve as scaffolds for new bone formation. Their use avoids the complications associated with autograft harvesting and the ethical concerns of xenogeneic and allogeneic sources [7, 8]. Despite these advantages, debate persists over their effectiveness in achieving long-term osseointegration and stable implant outcomes compared to biologic grafts.

The performance of various graft materials used in sinus augmentation has been assessed and compared histologically, radiographically, and clinically in numerous studies. However, existing literature is heterogeneous, and a clear consensus on the optimal graft material—particularly regarding synthetic options—remains elusive [9].

Therefore, the objective of this systematic review was to critically assess and compare the efficacy of alloplastic grafts against other commonly used graft materials in maxillary sinus lift procedures. Specifically, the review aimed to evaluate outcomes such as new bone formation, graft resorption, implant survival rates, and postoperative complications.

### Methodology

This review was carried out as per the Preferred Reporting Items for Systematic review and Meta-Analysis (PRISMA) statement [10]. and registered in Prospective Registration of Systematic Review (PROSPERO)- CRD420261351944.

The focused research question proposed was *“What is the efficacy of alloplastic grafts used in maxillary sinus augmentation in terms of new bone formation, graft resorption, and implant survival rates?”*. The Criteria for PICO in this review were as follows:

- Population: Patients undergoing maxillary sinus floor augmentation.
- Intervention: Alloplastic grafts.
- Comparison: Studies comparing other grafts. (autografts, xenografts, allogenic grafts)
- Outcomes: Histomorphometry, radiographic analysis, implant survival, complications.

### Eligibility criteria

Inclusion criteria:

- Human clinical studies (RCTs, cohort studies). Different study designs were included to capture both high-level evidence from RCTs and real-world clinical insights from cohort studies and case series. This approach enhances external validity and compensates for limited randomized data in MSFA research.
- Studies comparing at least two different graft materials.
- Articles reporting histomorphometric, radiographic, or implant survival data.
- English-language publications (Published from January 2010 up to 2025).

Exclusion criteria:

- Animal studies, in vitro research, or review articles without original data, Case reports, editorials, or conference abstracts.
- Case Series and Studies without a comparator or compared with alloplastic mixtures alone.
- Studies with unclear methodology or missing outcome data.
- Publications not available in English or lacking an English-language version

### Search strategy

A comprehensive literature search was conducted using the databases PubMed, Scopus, Web of Science, Embase, EBSCO, Google Scholar, Clinical trials.gov, and Cochrane to identify relevant studies on maxillary sinus augmentation using various bone graft materials. The search included articles published from January 2010 to December 2025. The following terms were used in various combinations with Boolean operators (“AND”, “OR”): “maxillary sinus augmentation,” “sinus lift,” “bone graft,” “autograft,” “allograft,” “xenograft,” “alloplast,” “hydroxyapatite,” “β-tricalcium phosphate,” “histomorphometric,” “bone regeneration,” “implant survival,” and “graft resorption.” Controlled vocabulary, such as MeSH terms in PubMed and Emtree terms in Scopus, was also incorporated to enhance search sensitivity. (Table 1)

**Table 1:** Search string for the study.

Database	Search string	*N*
PubMed	(“maxillary sinus augmentation” OR “sinus floor elevation” OR “sinus lift”) AND (“alloplastic graft” OR “synthetic graft” OR “hydroxyapatite” OR “bioactive glass” OR “calcium phosphate” OR “nanohydroxyapatite”) AND (“autograft” OR “xenograft” OR “allograft” OR “bone graft material”) AND (“histomorphometry” OR “radiographic analysis” OR “bone formation” OR “graft resorption” OR “implant survival” OR “implant success” OR “complications”)	21
	(maxillary sinus augmentation) AND (alloplastic grafts)	18
Scopus	TITLE-ABS-KEY ((“maxillary sinus augmentation” OR “sinus floor elevation” OR “sinus lift”) AND (“alloplastic graft*” OR “synthetic graft*” OR hydroxyapatite OR “bioactive glass” OR “calcium phosphate” OR nanohydroxyapatite) AND (autograft* OR xenograft* OR allograft* OR “bone graft material*”) AND (histomorphometry OR “radiographic analysis” OR “bone formation” OR “graft resorption” OR “implant survival” OR “implant success” OR complication*))	58
Web of Science	TS = ((“maxillary sinus augmentation” OR “sinus floor elevation” OR “sinus lift”) AND (“alloplastic graft*” OR “synthetic graft*” OR hydroxyapatite OR “bioactive glass” OR “calcium phosphate” OR nanohydroxyapatite) AND (autograft* OR xenograft* OR allograft* OR “bone graft material*”) AND (histomorphometry OR “radiographic analysis” OR “bone formation” OR “graft resorption” OR “implant survival” OR “implant success” OR complication*))	79
EBSCO	(“maxillary sinus augmentation” OR “sinus floor elevation” OR “sinus lift”) AND (“alloplastic graft*” OR “synthetic graft*” OR hydroxyapatite OR “bioactive glass” OR “calcium phosphate” OR nanohydroxyapatite) AND (autograft* OR xenograft* OR allograft* OR “bone graft material*”) AND (histomorphometry OR “radiographic analysis” OR “bone formation” OR “graft resorption” OR “implant survival” OR “implant success” OR complication*)	43
EMBASE	(‘maxillary sinus augmentation’:ti,ab,kw OR ‘sinus floor elevation’:ti,ab,kw OR ‘sinus lift’:ti,ab,kw) AND (‘alloplastic graft*‘:ti,ab,kw OR ‘synthetic graft*‘:ti,ab,kw OR hydroxyapatite:ti,ab,kw OR ‘bioactive glass’:ti,ab,kw OR ‘calcium phosphate’:ti,ab,kw OR nanohydroxyapatite:ti,ab,kw) AND (autograft*:ti,ab,kw OR xenograft*:ti,ab,kw OR allograft*:ti,ab,kw OR ‘bone graft material*‘:ti,ab,kw) AND (histomorphometry:ti,ab,kw OR ‘radiographic analysis’:ti,ab,kw OR ‘bone formation’:ti,ab,kw OR ‘graft resorption’:ti,ab,kw OR ‘implant survival’:ti,ab,kw OR ‘implant success’:ti,ab,kw OR complication*:ti,ab,kw)	29
Google Scholar	(“maxillary sinus augmentation”) AND (“alloplastic grafts”)	226
	(“maxillary sinus augmentation” OR “sinus floor elevation” OR “sinus lift”) AND (“alloplastic graft” OR “synthetic graft” OR “hydroxyapatite” OR “bioactive glass” OR “calcium phosphate” OR “nanohydroxyapatite”) AND (“autograft” OR “xenograft” OR “allograft” OR “bone graft material”)	1048
Clinical trials.gov	maxillary sinus augmentation AND graft	44
Cochrane	(maxillary sinus augmentation) AND (alloplastic grafts)	14

### Screening process

**Fig. 1:**
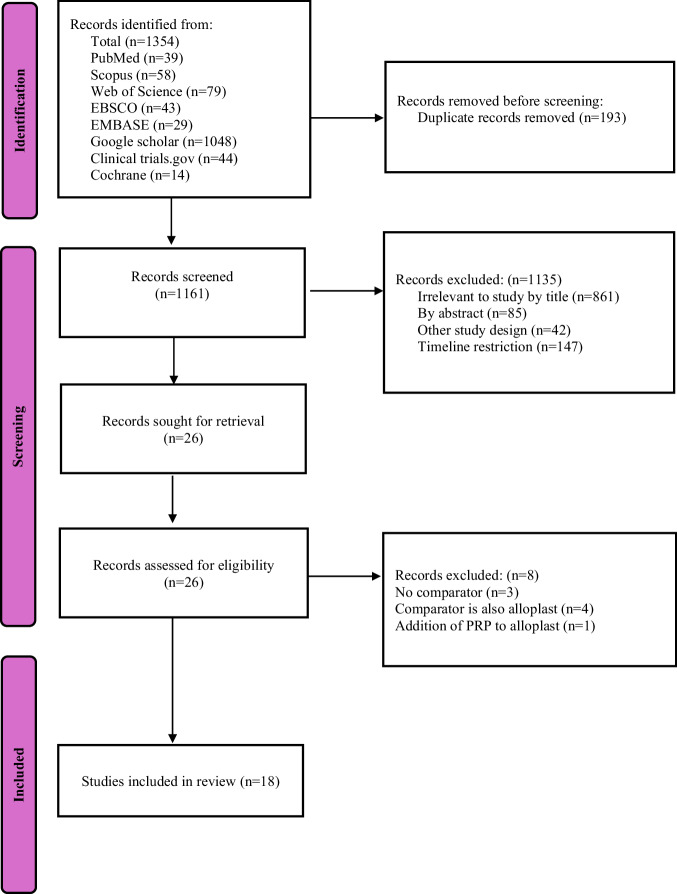
PRISMA Flowchart showing the study selection workflow.

The screening process followed a two-phase approach in accordance with PRISMA guidelines. In the first phase, two independent reviewers (A and B) screened the titles and abstracts of all retrieved articles based on the inclusion and exclusion criteria. Articles that appeared relevant or required further clarification were passed to the second phase for full-text evaluation. Discrepancies between reviewers were resolved through discussion, and a third reviewer (C) was consulted when consensus could not be reached. The study selection workflow is summarized in the PRISMA 2020 diagram (Fig. 1).

### Data extraction

Two reviewers (A and B) independently assessed relevance. Disagreements were resolved by a third reviewer (C). Data were extracted using a standardized form developed and piloted on a subset of studies. The following parameters were recorded: author, year of publication, study design, sample size, follow-up duration, graft material type (autogenous, allograft, xenograft, or alloplast), histomorphometric outcomes (percentage of new bone formation and residual graft), radiographic changes (bone height gain, volumetric stability), and implant success or survival rates. Where possible, numeric data were harmonized for consistency (e.g., percentages, millimeters). When essential data were missing, attempts were made to contact study authors.

### Risk of bias assessment of included studies

The methodological quality of the included studies was assessed based on the type of study design. For randomized controlled trials (RCTs), the Cochrane Risk of Bias tool (RoB 2.0) was utilized, evaluating five key domains, and for Non-randomized clinical trials, ROBINS I V2 was utilized, using six key domains [11, 12].

### Certainty of evidence

Certainty of evidence was assessed using the GRADE (Grading of Recommendations Assessment, Development, and Evaluation) system, with randomized trials starting as high certainty and observational studies as low certainty, and ratings adjusted based on predefined domains. This is based on Murad et al. [13] guidance for qualitative syntheses, ensuring transparent ratings even when meta-analysis could not be conducted.

## Results

### Study selection

A total of 1354 records were identified through eight database searches. After the removal of 193 duplicates, 1161 records were screened for relevance. Of these, 1135 were excluded following title review (*n* = 861), abstract screening (*n* = 85), study design incompatibility (*n* = 42), or failure to meet timeline restrictions (*n* = 147). Twenty-six full-text articles were retrieved for detailed eligibility assessment. Eight were excluded at this stage: three lacked a comparator, four used comparators that were also alloplasts, and one combined platelet-rich plasma (PRP) with alloplasts. Ultimately, 18 studies met all inclusion criteria and were incorporated into the systematic review [14–31]. A flowchart of the identification, inclusion, and exclusion of studies is shown in Fig. 1.

### Study characteristics

A total of 18 studies met the eligibility criteria and were included in the qualitative synthesis. These studies encompassed a mix of randomized controlled trials (*n* = 13) [14–26]. and non-randomized studies (*n* = 5) [27–31]. conducted across various clinical settings between 2010 and 2025. Sample sizes ranged from 5 to 56 patients per study, with follow-up durations varying from 5 months to 2 years. The graft materials investigated included autogenous bone, allografts (freeze-dried bone allograft (FDBA), Mineralized cortical bone allograft (MCBA)), xenografts (Deproteinized Bovine Bone Mineral (DBB/DBA/DPBM), Anorganic Bovine Bone), alloplasts (such as hydroxyapatite, β-tricalcium phosphate, Biphasic Calcium Phosphate, and Bioactive glass), and composite grafts combining two or more materials. Most studies employed the lateral window sinus lift technique, with or without adjunctive membrane use. Common outcome measures included histomorphometric parameters (new bone formation and residual graft percentage), radiographic changes (bone height gain and graft volume stability), and implant survival or success rates. Due to substantial heterogeneity in interventions, differences in follow-up timelines, and outcome reporting, a meta-analysis was not feasible. Consequently, a qualitative synthesis was undertaken to summarize the findings. The detailed study characteristics are presented in Tables 2 and 3.

**Table 2:** Study characteristics and outcomes of the included studies.

Author, year	Study design	Study setting	Sample size and allocation	Mean age	Gender distribution F:M	Intervention groups	Graft type	Follow-up duration	Outcomes	Key findings
**Lindgren et al**.[28].	Prospective study	Sweden	11 patients (22 sinuses, 62 implants)	67	6:5	BCP (HA/β-TCP 60/40, BoneCeramic), DBB (Bio-Oss)	Alloplast, xenograft	8 months healing, 1year post-loading follow-up	Implant survival/success, marginal bone loss, graft resorption, peri-implant parameters (PLI, BPI, SBI, PPD)	Implant survival was high and similar for both biomaterials. Success rates were slightly higher with DBB, but differences were not significant. Marginal bone loss and graft resorption were comparable between groups. Both grafts supported stable peri-implant tissues and reliable implant integration after sinus augmentation.
**Kurkcu et al**. [30].	Comparative clinical study	Turkey	23 patients (23 sinus augmentation, 51 implants)	48.6	12:11	Anorganic BHA(BonePlus-xs), ß-TCP (Kasios TCP)	Xenograft, Alloplast	6–8 months healing	Histomorphometry: new bone %, residual graft %, soft tissue %	Both materials biocompatible and osteoconductive, but BHA more efficient in osteoconduction.
**Schmitt et al.** [17].	RCT	Germany	30 patients (45 sinuses, 94 implants)	Not explicitly stated	17:13	BCP (BoneCeramic), ABB (Bio-Oss), MCBA (Puros), AB	Alloplast, Xenograft, Allograft, Autograft	5 months	Histomorphometry: new bone %, residual graft %, mineralized vs. soft tissue fractions	Autogenous bone yielded the highest new bone formation. MCBA showed results close to autogenous bone, while BCP and ABB produced lower but acceptable levels of new bone. Residual graft material was higher in ABB compared to BCP, indicating slower resorption. All substitutes demonstrated good tissue integration and were suitable for sinus augmentation, though autogenous bone remained superior in regenerative potential.
**de Lange et al.** [29].	Split-mouth study	Netherlands	5 edentulous patients (bilateral sinus augmentation, 32 implants)	66	4:1	BCP (Straumann BoneCeramic, HA/TCP 60/40), DBA (Bio-Oss)	Alloplast, Xenograft	3–8 months healing, 4-year implant follow-up	Histomorphometry + micro-CT: bone volume %, graft volume %, mineralization, osteoid %, osteoclast/osteocyte counts; implant survival, peri-implant mucosa health	Both grafts effective for sinus augmentation and implant placement. Bone volume and mineralization similar. BCP biopsies showed more osteoid and signs of active remodeling. Clinically, implant survival and peri-implant mucosa health were comparable, confirming both materials as effective sinus graft substitutes
**Mordenfeld et al.** [25].	RCT	Sweden	11 patients (bilateral sinus augmentation, 62 implants)	67	6:5	BCP (BoneCeramic 60/40 HA/TCP), DBB (Bio-Oss)	Alloplast, xenograft	8 months healing, 5 year post-loading follow-up	Implant survival/success, marginal bone loss, grafted sinus height reduction (GHR)	Both grafts supported high implant survival and stable prosthetic function over 5 years. Survival rates were nearly identical between BCP and DBB, with no significant differences in marginal bone loss. Success rates were slightly higher with DBB, but differences were not statistically significant. Graft height reduction was minimal and comparable between groups, confirming long-term volumetric stability. Both biomaterials proved effective and reliable for sinus augmentation, with outcomes sustained over extended follow-up.
**De.O.Gorla et al.** [14].	Randomized volumetric CBCT study	Brazil	22 patients (36 sinuses)	Not explicitly stated	16:6	AB, β-TCP, AB + β-TCP	Autograft, Alloplast, Composite	6 months post-MSFA	Volumetric bone resorption (CBCT), graft volume maintenance	Autogenous bone, autogenous mixed with β-TCP, and β-TCP alone all provided acceptable outcomes. Autogenous bone resorbed the most, while β-TCP alone demonstrated the best volumetric stability, suggesting it can serve as a safe substitute without donor-site morbidity.
**Onișor-Gligor et al.** [27].	Bilateral comparative study	Romania	21 patients (bilateral sinus lifts)	Women: 53.55 Men: 61.00	11:10	Iliac crest bone, PerioGlass	Autograft, Alloplast	24 months post-MSFA	Implant failure rate, bone resorption at 6, 12, 24 months, post-loading resorption	Autologous grafts yielded lower implant failure but showed greater resorption over time. Alloplastic grafts were more stable volumetrically, though implant failures were slightly higher. Both materials achieved high overall implant survival, confirming their clinical utility.
**Ahmet et al.** [15].	Randomized controlled trial	Istanbul	20 patients → 16 completed (13 CA, 10 CB)	53.8	8:12	BCP + DBB, BCP + HA/β-TCP	Xenograft, Alloplast	5 months post-MSFA	New bone formation, residual graft, osteoconduction, graft height stability	Both composite grafts were biocompatible and effective for sinus augmentation. Bone formation was similar between the two groups, but the mixture with bovine bone maintained graft height more reliably than the mixture with synthetic alloplast.
**Danesh-Sani et al.** [26].	RCT	USA, Italy	10 patients (bilateral sinus augmentation)	Not explicitly stated	Not explicitly stated	Autogenous bone chips (zygomatic buttress, lateral sinus wall, tuberosity), BCP (Straumann Bone Ceramic, 60% HA/40% ß-TCP)	Autograft, Alloplast	6–8 months	Histomorphometry: new bone %, residual graft %, soft tissue %; Clinical implant survival	Both grafts were effective and biocompatible for sinus augmentation. AB produced significantly more vital bone and showed minimal residual graft, but it also presented higher proportions of soft tissue. BCP demonstrated good osteoconductivity, with residual particles persisting longer and less vital bone compared to autogenous grafts. Despite these differences, both materials achieved successful clinical outcomes, confirming BCP as a reliable substitute when autogenous bone is not preferred.
**Kolerman et al.** [16].	Randomized split-mouth study	Israel	13 patients (26 sinuses)	58	7:6	FDBA, BCP	Allograft, Alloplast	9 months post-MSFA	Histomorphometry (new bone %, residual graft %, osteoconduction), inflammation, bone-to-graft contact	Both allograft and alloplast supported new bone formation to a comparable degree. The allograft showed less residual graft material and stronger osteoconductive integration, while the alloplast exhibited more residual particles and a mild inflammatory response
**Bonardi et al.** [31].	Prospective clinical study	Brazil	30 patients (30 sinuses)	Not explicitly stated	Not explicitly stated	AB, ChronOS (ß-TCP), ChronOS+AB, Bio-Oss (DBBM), Bio-Oss+AB	Autograft, Alloplast, Xenograft, Composite	6 months	Histomorphometry (new bone, residual biomaterial, connective tissue); Immunohistochemistry (Runx2, VEGF, osteocalcin)	ChronOS+AB similar to autogenous bone; Bio-Oss groups showed more residual biomaterial but stronger maturation signals; ChronOS alone yielded more new bone than Bio-Oss alone.
**Pereira et al.** [24].	RCT	Brazil	22 patients (36 sinuses)	Not explicitly stated	Not explicitly stated	ß-TCP alone, ß-TCP+autogenous bone (1:1), AB	Alloplast, composite, autograft	6 months	Histomorphometry: new bone % (Pristine/Intermediate/Apical); Immunohistochemistry: RUNX2, VEGF	All grafts produced acceptable bone formation. β-TCP alone behaved similarly to autogenous bone, showing mature bone and stable outcomes. The composite group (β-TCP + AB) demonstrated delayed maturation, with higher cellular activity and immature bone. Mixing AB with β-TCP did not improve results and instead slowed maturation. β-TCP alone was considered a safe substitute, comparable to AB, while composites offered no added benefit.
**Chiu et al.** [23].	RCT	USA	13 patients (bilateral sinus augmentation), 26 microimplants	60.6	5:8	Osteon (synthetic BCP, HA/TCP 70/30) vs. Bio-Oss (DBBM xenograft)	Alloplast, xenograft	6–8 months	Histomorphometry: % bone, marrow/fibrous tissue, residual graft; Bone-to-implant contact (BIC)	Both grafts demonstrated comparable new bone formation and successful osseointegration. Bone-to-implant contact was similar overall, but synthetic BCP showed significantly higher contact in the coronal threads, suggesting more active early integration. No inflammation was observed in either group. Clinically, both grafts were effective, with synthetic alloplast performing equivalently to xenograft, supporting its use as a viable alternative.
**Oh et al.** [22].	RCT	Republic of Korea	56 patients (60 sinuses)	54.3	25:33	BCP (HA/β-TCP 60/40, Osteon III), DBBM (Bio-Oss)	Alloplast, xenograft	6 months post augmentation and mean implant follow-up up to 21 months	Micro-CT (new bone %, residual graft %, bone surface density), histomorphometry (new bone %, marrow space), implant stability (ISQ)	Both grafts showed similar biocompatibility and osteoconductivity; BCP tended toward higher new bone volume and surface density, DBBM toward higher residual graft volume. No significant differences in histomorphometric or micro-CT outcomes
**Kolerman et al.** [21].	Randomized split-mouth study	Israel	13 patients (26 sinuses)	57.8	7:6	FDBA, BCP (BCP, 60% HA/40% ß‑TCP)	Allograft, Alloplast	9 months post-MSFA	Histomorphometry: gradient of new bone (NB) and residual graft particles	Both materials effective, but FDBA showed lower residual graft and more consistent NB formation at greater distances. Osteogenesis gradient confirmed, highlighting the importance of proximity to native bone.
**Kraus et al.** [20].	RCT	Switzerland, Germany	56 patients	59.3 years	37:19	(BCP, HA/TCP) 10:90, Straumann (VivOss), (DBBM, Bio-Oss)	Alloplast, xenograft	6 months	Histomorphometry: new bone %, residual graft %, non-mineralized tissue %; Implant survival/success	Both grafts produced similar amounts of new bone after sinus augmentation. BCP showed less residual graft material and more soft tissue compared with DBBM, indicating faster remodeling. Implant survival was high in both groups, with no significant differences. Clinically, both materials were effective, but BCP demonstrated more active turnover while DBBM provided slower resorption and long-term stability.
**Arunjaroensuk et al.** [19].	RCT	Thailand	30 specimens	55.77	11:19	(BCP, HA/β-TCP ratio 70/30), DBBM	Alloplast, xenograft	6–10 months post augmentation	Micro-CT (BV/TV, GV/TV), histology (new bone %, residual graft %), gene expression (ALP, OSX, IL-1B, TRAP)	Both grafts produced similar outcomes in terms of new bone formation and residual graft particles, with no significant differences in histology or micro-CT. Gene expression patterns showed increased osteogenic and osteoclastic activity in augmented bone compared with native bone, but again no differences between BCP and DBBM. The only notable finding was a higher osteoclastic marker expression with BCP, suggesting more active remodeling, though clinically both materials were equivalent.
**Schmitt et al.** [17].	RCT	Germany	24 patients (27 sinuses)	50.9	15:9	DBBM (Bio-Oss), DPBM (The Graft), BCP (Osopia, TCP/HA > 90/10)	Xenograft, Alloplast	4–6 months healing	Graft volume stability (VOL%, VOLmm³), MeanD, MaxD, MinD	DBBM and DPBM maintained stable graft volumes after healing, confirming their long-term dimensional stability. In contrast, BCP showed significant resorption and reduced graft volume, indicating inferior volumetric stability compared with the xenografts. Despite this, implants could still be placed successfully in all groups, but the synthetic material demonstrated less predictable volume maintenance.

*AB* Autologous bone, *BHA* Bovine-derived hydroxyapatite, *BCP* Biphasic Calcium Phosphate, *HA* Hydroxyapatite, *β-TCP* Beta-Tricalcium Phosphate, *DBB/DBBM/DBA* Deproteinized Bovine Bone, *ABB* Anorganic Bovine bone, *MCBA* Mineralized cortical bone allograft, *FDBA* Freeze-dried bone allograft, *DPBM* Deproteinized Porcine Bone mineral, *BIC* Bone-to-implant contact.

**Table 3:** Graft-specific outcomes across included studies.

Study (Year)	Outcome	Alloplast	Xenograft	Allograft	Autograft
Lindgren et al. [28].	Implant survival	96.8%	96.8%		
	Success rate	91.7%	95.7%		
	Marginal bone loss (mm)	0.43 ± 0.20	0.29 ± 0.10		
	Graft resorption (mm)	0.43	0.29		
Kurkcu et al. [30].	New bone (%)	21.09 ± 2.86	30.13 ± 3.45		
	Residual graft (%)	34.05 ± 3.01	31.88 ± 6.05		
Schmitt et al. [18].	New bone (%)	30.28 ± 2.16	24.9 ± 5.67	35.41 ± 2.78	41.74 ± 2.1
	Residual graft (%)	15.8 ± 2.1	21.36 ± 4.83		
de Lange et al.[29].	Bone height gain	21.8	100%		
	Implant survival	15/16	21.2		
Mordenfeld et al.[25].	Implant survival	91.7%	91.3%		
	Marginal bone loss (mm)	1.4 ± 1.2	1.0 ± 0.7		
	Implant success	83.3%	91.3%		
Gorla et al. [14].	Graft volume loss (%)	38.33 ± 16.64	-		45.75 ± 18.65
Onişor-Gligor et al.[27].	Implant failure rate (%)	7.69	-		1.89
	BR1/BR2/BR3/BRIL (%)	3.66/ 9.87/15.49/ 6.17	-		7.40/18.87/25.39 /8.05
Danesh-Sani et al.[26].	New bone (%)	28.2 ± 8.4	-		36.8 ± 11.5
	Residual graft (%)	32.9 ± 8.1	-		4.8 ± 2.4
	Implant survival	100%	-		
Ahmet et al. [15]. – Group CA	New bone (%)	34.40 ± 18.91	-	36.71 ± 15.32	
	Residual graft (%)	6.98 ± 5.09	-	5.52 ± 4.12	
	Graft height loss (mm / %)	4.14 ± 0.58 / 24.44 ± 6.52	-	2.52 ± 0.67 / 14.60 ± 4.58	
Kolerman et al.[16].	New bone (%)	24.0 ± 6.8	-	27.5 ± 8.1	
	Residual graft (%)	25.4 ± 5.5	-	12.5 ± 8.1	
	Osteoconductive value (%)	26.7 ± 7.9	-	52.6 ± 16.9	
Bonardi et al. [31].	New bone formation (µm²)	83,787	65,717		121,917
	Remaining biomaterial (µm²)	5291.0	56,258.5		
Pereira et al. [24].	New bone (%) (pristine/intermediate/apical)	46.3/ 47.6/ 44.8	-		43.1/ 31.0/ 46.1
Chiu et al. [23].	Vital bone (TImpC) (%)	13.35 ± 4.01	13.35 ± 4.83		
Oh et al. [22].	New bone (%)	28.84 ± 7.94	6.17% ± 3.73		
	Residual graft volume (%)	26.99% ± 9.16	25.13 ± 9.56		
	Implant survival (%)	100	32.19 ± 11.73		
Kolerman et al.[21].	New bone (%)	30.0 ± 11.0 (G1), 23.5 ± 9.9 (G2)	31.0 ± 9.5 (G1), 27.7 ± 11.2 (G2)		
	Residual graft (%)	21.9 ± 9.9 (G1), 27.7 ± 6.6 (G2)	7.1 ± 6.6 (G1), 9.1 ± 10.3 (G2)		
Kraus et al.[20].	Newly formed bone (%)	35.9	100		
	Patient-level implant survival (%) Implant level survival (%)	96 96.9	88.8 94.7		
Arunjaroensuk et al. [19].	New bone (%)	31.43 ± 14.36	30.09 ± 9.86		
Schmitt et al. [17].	Volumetric stability (%)	66 ± 25	103 ± 4	112 ± 23	
	Mean graft volume (mm³)	617.93 ± 168.98	1424.45 ± 780.70	1130.80 ± 352.53	

### Risk of bias and certainty of evidence

The risk of bias across the included studies varied depending on study design and methodological quality. Among the thirteen RCTs, two were assessed as having a high risk of bias primarily due to randomization and allocation concealment (21,22). Two studies demonstrated low risk of bias [20, 26], and nine studies were assessed as some concerns [14–19, 23–25]. All five non-randomized studies exhibited some concerns due to confounding factors [27–31]. The distribution of bias assessments is summarized in Table 4.

**Table 4:** Risk of bias assessment of included studies using the Cochrane risk of bias tool.

Cochrane risk of bias for randomized studies (RoB 2)							
Author, Year	D1	D2	D3	D4	D5	OVERALL	
Schmitt et al. [18].	Some concerns	Some concerns	Low	Some concerns	Low	Some concerns	
Mordenfeld et al. [25].	Some concerns	Some concerns	Low	Some concerns	Low	Some concerns	
Danesh-Sani et al. [26].	Low	Low	Low	Low	Low	Low	
Pereira et al. [24].	Some concerns	Low	Low	Some concerns	Low	Some concerns	
Chiu et al. [23].	Some concerns	Low	Low	Low	Low	Some concerns	
Oh et al. [22].	High	Some concerns	Low	Some concerns	Low	High	
Kolerman et al. [21].	High	Some concerns	Low	Some concerns	Low	High	
Kraus et al. [20].	Low	Low	Low	Low	Low	Low	
Arunjaroensuk et al. [19].	Low	Low	Low	Some concerns	Low	Some concerns	
Schmitt et al. [17].	Low	Low	Low	Some concerns	Low	Some concerns	
De O Gorla et al. [14].	Some concerns	Low	Low	Some concerns	Low	Some concerns	
Ahmet et al. [15].	Some concerns	Some concerns	Low	Some concerns	Low	Some concerns	
Kolerman et al. [16].	Some concerns	Some concerns	Low	Some concerns	Low	Some concerns	
**Cochrane risk of bias for non-randomized studies (ROBINS I)**							
**Author, Year**	**D1**	**D2**	**D3**	**D4**	**D5**	**D6**	**Overall**
Lindgren et al. [28].	Some concerns	Low	Low	Low	Some concerns	Low	Some concerns
de Lange et al. [29].	Some concerns	Low	Low	Low	Some concerns	Low	Some concerns
Kurkcu et al. [30].	Some concerns	Low	Low	Some concerns	Some concerns	Low	Some concerns
Bonardi et al. [31].	Some concerns	Low	Low	Low	Some concerns	Low	Some concerns
Onisor-Gligor et al. [14].	Some concerns	Low	Low	Low	Some concerns	Low	Some concerns

The certainty of evidence for new bone formation, graft resorption, and implant survival following maxillary sinus augmentation with alloplastic grafts was graded as low. The detailed characteristics are represented in Table 5.

**Table 5:** GRADE assessment of the included studies.

Outcomes assessed	Studies	Reasons for downgrading the evidence	Quality of evidence
Efficacy of alloplastic grafts used in maxillary sinus augmentation in terms of new bone formation, graft resorption, and implant survival rates	Schmitt et al. [18]. Ahmet et al. [15]. Danesh-Sani et al. [26]. Pereira et al. [24]. Chiu et al. [23], Oh et al.[22]. Kraus et al. [22]. Arunjaroensuk et al. [19].	Downgrading factors: (−2) RoB: (−1) Serious Included RCT were with high Rob (*n* = 2), some concerns (*n* = 9) and low (*n* = 2) based on Cochrane RoB 2.0 Indirectness: Not Serious As some heterogeneity in primary outcome variable, study setting, timeline Imprecision: (−1) Serious Imprecision was judged qualitatively due to the relatively small cumulative sample size across the included trials. Confidence interval–based assessment was not feasible as meta-analysis was not performed. Inconsistency: Not Serious Publication bias: Not detected formally	Low ⊕⊕⊖⊖

### Qualitative analysis

#### Histomorphometric Outcomes

New bone formation (NBF) varied across graft types, with autogenous bone generally demonstrating the highest values. In Ahmet et al. [15] study, both groups-calcium sulfate (CS) combined with alloplast and CS combined with DBB, demonstrated comparable new bone formation (34.4% vs. 36.7%, respectively). However, residual graft particles were slightly higher in the alloplast group (6.98%) compared to DBB (5.52%), suggesting a marginally slower resorption profile for the synthetic material. Schmitt et al. [18]. reported NBF of 41.7% with autografts, compared to 35.4% for allografts, 30.3% for alloplasts, and 24.9% for xenografts. Danesh-Sani et al. [26] corroborated this trend, showing greater NBF in autografts (36.8%) compared to alloplasts (28.2%). Residual graft content was consistently higher in synthetic groups: Schmitt et al. [18] observed 15.8% residual particles in alloplasts versus 21.4% in xenografts, while Danesh-Sani et al. reported 32.9% residual content in alloplasts compared to only 4.8% in autografts. Kolerman et al. [16] further highlighted this difference, with BCP (alloplast) retaining 25.4% residual graft versus 12.5% in FDBA (allograft), alongside a markedly lower osteoconductive value (26.7% vs. 52.6%). These findings suggest that biologic grafts integrate more efficiently, while synthetic substitutes persist longer, contributing to dimensional stability but slower remodeling.

#### Volumetric and radiographic outcomes

Volumetric stability favored xenografts and synthetics. Schmitt et al. [17] reported volumetric stability of 103% in xenografts and 112% in allografts, compared to 66% in alloplasts. Gorla et al. [14] observed greater graft volume loss in autografts (45.8%) compared to alloplasts (38.3%), indicating that synthetic materials may preserve dimensions more effectively. Ahmet et al. [15] found graft height loss was significantly higher in the alloplast group (4.14 mm, 24.4%) compared to xenografts (2.52 mm, 14.6%), reinforcing the superior space maintenance of DBBM. Radiographic assessments by de Lange et al. [29] demonstrated complete bone height gain in xenografts, while alloplasts achieved only partial gains (21.8%), underscoring the dimensional reliability of biologic substitutes.

#### Implant survival and clinical outcomes

Implant survival rates were consistently high across graft types, typically exceeding 90%. Lindgren et al. [28] reported identical survival rates for alloplasts and xenografts (96.8%), though success rates were slightly higher in xenografts (95.7% vs. 91.7%). Mordenfeld et al. [25] found comparable survival (91.7% alloplast vs. 91.3% xenograft), but marginal bone loss was lower in xenografts (1.0 mm vs. 1.4 mm). Danesh-Sani et al. [26] reported 100% survival in alloplasts, while Onișor-Gligor et al. [27] observed higher implant failure in alloplasts (7.7%) compared to autografts (1.9%). Kraus et al. [20] confirmed excellent survival across groups, with patient-level survival of 96% for alloplasts and 88.8% for xenografts. These findings suggest that while survival rates are broadly comparable, biologic grafts may confer marginal advantages in long-term success and reduced failure rates.

## Discussion

This systematic review evaluated the efficacy of alloplastic grafts in maxillary sinus augmentation, focusing on key outcomes including new bone formation, graft resorption, and implant success. The evidence indicates that alloplastic materials such as hydroxyapatite (HA), β-tricalcium phosphate (β-TCP), and biphasic calcium phosphate (BCP) are biocompatible and osteoconductive, consistently supporting favorable implant survival rates.

However, their biological behavior differs from that of autografts and xenografts, particularly with respect to remodeling kinetics and volumetric stability. While alloplastic grafts demonstrated comparable clinical performance to other graft types in terms of implant survival and low complication rates, autogenous bone grafts tended to exhibit greater new bone formation during the early healing phase, albeit with increased donor-site morbidity and a higher risk of resorption.

Autogenous bone consistently produced the highest percentages of vital bone in the early healing phase, as shown by Schmitt et al. [17, 18] and Danesh-Sani et al. [26]. This superiority is explained by the presence of living osteogenic cells and growth factors in autografts, which accelerate osteogenesis [32, 33].

In contrast, alloplasts such as BCP and β-TCP yielded slightly lower amounts of vital bone, often accompanied by more residual particles [16]. The persistence of residual particles reflects slower remodeling, but this scaffold effect ensures dimensional support during healing. Pereira et al. substantiated that β-TCP alone behaved similarly to autogenous bone in terms of bone quality, producing mature bone and stable outcomes. Chiu et al. further demonstrated that synthetic BCP achieved bone-to-implant contact equivalent to DBBM, with even higher contact in coronal threads, suggesting that synthetics may promote more active early integration at the implant interface. Thus, while autografts remain biologically superior, alloplasts reliably support osteogenesis sufficient for implant placement, with the slower turnover explained by their purely osteoconductive nature rather than osteoinductive potential.

Resorption behavior is critical in sinus augmentation, where dimensional stability under pneumatic sinus pressure is essential. Gorla et al. [14] reported that β-TCP alone maintained graft volume better than autogenous or composite grafts, highlighting the role of synthetics in space maintenance. This stability is explained by the slower resorption of β-TCP compared to autogenous bone, which undergoes rapid remodeling and volume loss. Mordenfeld et al. [25] confirmed minimal graft height reduction with BCP over five years, comparable to DBBM, underscoring long-term dimensional reliability. However, Schmitt et al. [18] provided three-dimensional volumetric evidence that BCP resorbed significantly more than DBBM and DPBM, which maintained stable graft volumes. This difference is explained by the higher proportion of resorbable TCP in the synthetic, which accelerates turnover but compromises volumetric predictability. Arunjaroensuk et al. [19] added molecular insights, showing similar histological and volumetric outcomes between BCP and DBBM, but with higher osteoclastic marker expression in BCP, indicating more dynamic remodeling. Onișor-Gligor et al. [27] reported that autologous grafts showed lower implant failure but greater resorption over time, whereas alloplasts were more stable volumetrically, albeit with slightly higher failure rates. Ahmet et al. [15] highlighted that composite grafts combining BCP with DBB maintained graft height more reliably than mixtures with synthetic alloplast alone, underscoring the role of xenografts in dimensional preservation. More recently, Kraus et al. [20] and Oh et al. [22] confirmed that BCP and DBBM produced similar new bone formation, but BCP showed less residual graft and more remodeling activity. This suggests that synthetics may favor biological turnover but at the expense of dimensional stability, whereas xenografts provide slower resorption and long-term scaffold persistence.

Across all studies, implant survival was consistently high, exceeding 90% regardless of graft type. Lindgren et al. [28] and Mordenfeld et al. [25] reported survival rates above 90% for both BCP and DBBM, with no significant differences in marginal bone loss or peri-implant tissue health. Danesh-Sani et al. [26] achieved 100% survival with both autogenous bone and BCP, despite differences in histomorphometry. Onișor-Gligor et al. [27] noted slightly higher implant failure rates with alloplasts compared to autografts, but these remained within acceptable thresholds, and importantly, the alloplast group showed lower long-term resorption. The consistently high success rates lie in the fact that implant osseointegration depends primarily on achieving sufficient vital bone at the implant interface, which alloplasts reliably provide, even if overall remodeling is slower.

The comparative histological evidence highlights consistent differences in the biological behavior of biphasic calcium phosphate (BCP/β-TCP) and deproteinized bovine bone mineral (DBBM), with autogenous bone (AB) serving as the benchmark for vital bone formation. BCP demonstrated more dynamic remodeling. Histology revealed woven bone formation, osteoclast-mediated resorption of graft particles, and ingrowth of new bone into micropores [19, 22]. The presence of osteoid in BCP biopsies [29] further supports the notion of ongoing active bone formation. Immunohistochemical analyses corroborated these findings: RUNX2 and VEGF expression were higher in BCP or BCP-based composites [19, 24]. indicating enhanced osteoblast differentiation and angiogenesis. These markers suggest that BCP accelerates the early phases of bone turnover, although the bone formed is often immature at the 6–9 month evaluation point.

In contrast, DBBM consistently acted as a stable scaffold. Histological sections showed residual particles largely maintaining their original form, embedded within new bone, with minimal evidence of resorption [21, 22]. While DBBM yielded comparable amounts of mineralized bone to BCP [20, 29]. The tissue dynamics differed: DBBM sites contained less osteoid and fewer signs of active remodeling, reflecting slower turnover. This stability may contribute to long-term dimensional maintenance, but at the expense of reduced biological activity.

Autogenous bone produced the highest fraction of vital lamellar bone with well-vascularized marrow spaces [24, 26]. However, its clinical limitations make substitutes necessary. Mixture of BCP with AB did not enhance outcomes; instead, it was associated with immature bone and delayed maturation [24], suggesting that small admixtures may dilute the osteogenic potential rather than augment it.

Alloplastic grafts were associated with low complication rates, with minimal risk of sinus membrane perforation or infection. Unlike autografts, they avoid donor-site morbidity, and unlike xenografts or allografts, they eliminate concerns related to immunogenicity or disease transmission [34]. These safety advantages make them particularly attractive for medically compromised patients or those requiring bilateral sinus lifts.

The present study correlates with the findings of Guruprasad et al. [35], who reported comparable new bone formation between allografts and other regenerative grafts, with higher residual graft content observed in the latter. These results align with our observations on alloplastic grafts, which demonstrated moderate bone regeneration and slower resorption kinetics relative to autogenous and xenograft materials. However, unlike Guruprasad et al. [35], our synthesis incorporated implant failure rates and volumetric stability, revealing slightly elevated failure rates and greater graft height loss with alloplasts. This divergence underscores the importance of evaluating both histologic and clinical endpoints when assessing graft performance in MSFA.

Thus, the use of alloplastic grafts can be considered as a viable alternative to autografts or xenografts in maxillary sinus augmentation, particularly in clinical scenarios where minimizing patient morbidity is a priority or where infection and immunological risks must be avoided. Their synthetic origin, sterility, and predictable degradation profile make them especially suitable in patients with systemic comorbidities or when donor-site harvesting is contraindicated. Moreover, their use may be advantageous in staged or delayed implant protocols, where extended healing periods allow for gradual scaffold remodeling.

However, clinicians should exercise caution when selecting alloplasts in cases requiring rapid bone turnover or early implant placement. In such contexts, autogenous grafts or biologically active composites may offer superior regenerative potential due to their inherent osteogenic and osteoinductive properties. Tailoring graft selection to the biologic demands of the site and the timing of implant placement remains essential for optimizing clinical outcomes.

This review is limited by the heterogeneity of included studies (e.g., different follow-up periods, graft materials, and outcome measures), relatively small sample sizes in many trials, and the lack of long-term (>5 year) data for alloplastic materials in the majority of the studies. Furthermore, the high risk of bias in included RCTs may limit internal validity. Future research should prioritize large-scale, well-designed randomized controlled trials (RCTs) employing standardized protocols to enable meaningful comparisons across graft materials. Longitudinal studies extending beyond 3–5 years are particularly needed to elucidate the long-term remodeling dynamics and volumetric stability of alloplastic grafts. Additionally, emerging innovations such as bioactive and nanostructured alloplastic materials warrant focused investigation, as they may offer enhanced osteointegration, accelerated remodeling, and improved clinical outcomes compared to conventional synthetic grafts.

## Conclusion

Alloplastic grafts represent a safe, biocompatible, and clinically effective alternative to biologic materials, particularly in patients where autograft harvesting is contraindicated or when minimizing morbidity and immunological risk is essential. Their predictable resorption profile and scaffold stability make them especially suitable for sinus augmentation and other procedures where volumetric maintenance is prioritized. Although their osteogenic and osteoinductive potential is inherently limited and they have inferior results in terms of regenerative potential when compared to biologic grafts, their performance can be optimized through composite grafting strategies or staged protocols. When selected appropriately, alloplasts offer a reliable solution with favorable implant survival and low complication rates. High-quality, long-term randomized trials are needed to confirm these findings and optimize graft selection.

## Data Availability

Data related to the included studies is drafted in the manuscript
